# Ebola Virus Glycoprotein Strongly Binds to Membranes in the Absence of Receptor Engagement

**DOI:** 10.1021/acsinfecdis.3c00622

**Published:** 2024-04-29

**Authors:** Alisa Vaknin, Alon Grossman, Natasha D. Durham, Inbal Lupovitz, Shahar Goren, Gonen Golani, Yael Roichman, James B. Munro, Raya Sorkin

**Affiliations:** † School of Chemistry, Raymond & Beverly Sackler Faculty of Exact Sciences, Tel Aviv University, Tel Aviv 6997801, Israel; ‡ Center for Physics and Chemistry of Living Systems, Tel Aviv University, Tel Aviv 6997801, Israel; § Department of Microbiology and Physiological Systems, University of Massachusetts Chan Medical School, Worcester, Massachusetts 01605, United States; ∥ Department of Physics and Haifa Research Center for Theoretical Physics and Astrophysics, University of Haifa, Haifa 3498838, Israel; ⊥ Raymond and Beverly Sackler School of Physics & Astronomy, Tel Aviv University, Tel Aviv 6997801, Israel; # Department of Biochemistry and Molecular Biotechnology, University of Massachusetts Chan Medical School, Worcester, Massachusetts 01605, United States

**Keywords:** Ebola virus, glycoprotein, fusogens, optical tweezers, particle tracking, DNA stretching

## Abstract

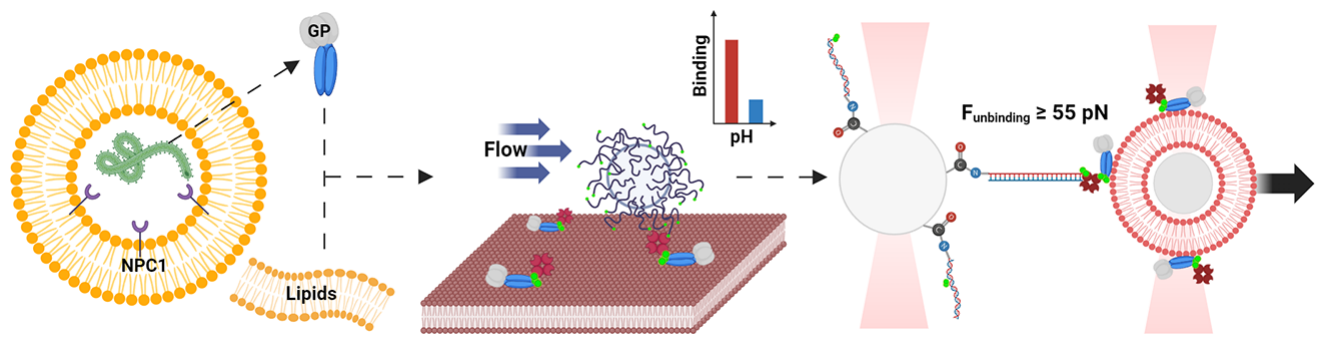

Ebola virus (EBOV) is an enveloped virus that must fuse with the host cell membrane in order to release its genome and initiate infection. This process requires the action of the EBOV envelope glycoprotein (GP), encoded by the virus, which resides in the viral envelope and consists of a receptor binding subunit, GP1, and a membrane fusion subunit, GP2. Despite extensive research, a mechanistic understanding of the viral fusion process is incomplete. To investigate GP-membrane association, a key step in the fusion process, we used two approaches: high-throughput measurements of single-particle diffusion and single-molecule measurements with optical tweezers. Using these methods, we show that the presence of the endosomal Niemann-Pick C1 (NPC1) receptor is not required for primed GP-membrane binding. In addition, we demonstrate this binding is very strong, likely attributed to the interaction between the GP fusion loop and the membrane’s hydrophobic core. Our results also align with previously reported findings, emphasizing the significance of acidic pH in the protein–membrane interaction. Beyond Ebola virus research, our approach provides a powerful toolkit for studying other protein–membrane interactions, opening new avenues for a better understanding of protein-mediated membrane fusion events.

The Ebola virus (EBOV) disease leads to severe symptoms such as hemorrhagic fever, with a fatality rate ranging from 25% to 90%.^1,2^ With the most recent outbreak having ended in January 2023, resulting in a 50% mortality,^3^ the need for improved therapeutics is undeniable. As there are many outstanding questions about the molecular processes of EBOV pathogenesis, gaining novel mechanistic insights into the nature of the EBOV host cell invasion process is crucial in achieving this goal.

The EBOV-cellular entry and fusion processes encompass a set of complex, not yet fully elucidated, interactions and cellular components. EBOV is an enveloped virus containing a class I viral fusion glycoprotein (GP).^4−6^ GP is a trimer of heterodimers, with each protomer formed by two disulfide-bridged protein subunits, GP1 and GP2.^5^ The GP1 subunit mediates the attachment to the host cell plasma membrane through nonspecific receptors, such as C-type lectins,^5^ while the plasma membrane penetration is facilitated by endocytosis.^5,7^ Within the endosome, the host cathepsin proteases, which are active at acidic pH, remove the glycan-rich mucin-like domain and the glycan cap,^6,8^ priming the GP1 subunit for interaction with its endosomal Niemann–Pick C1 (NPC1) receptor.^9,10^ This interaction is crucial for the subsequent fusion of viral and host endosomal membranes.^4,9−11^

The membrane fusion process mediated by GP is similar to that of other class I viral fusion proteins^5,6,12,13^ and is facilitated by the GP2 subunit. Each GP2 subunit harbors a fusion loop, which resides within a hydrophobic cleft in the neighboring protomer.^14^ First, the fusion loop becomes exposed,^5^ subsequently inserting into the host endosomal membrane.^15^ Finally, a conformational change within GP brings the membranes into proximity, overcoming the energy barriers for hemifusion and fusion pore formation.^16^ Expansion of the fusion pore releases the viral genome into the cytoplasm.

Despite the wealth of information about the EBOV infection cycle, several knowledge gaps remain. Here, we aimed to address some of the unresolved questions regarding the EBOV fusion mechanism. First, we tested whether membrane association can occur in the absence of NPC1. Specifically, we explored whether the interaction with NPC1 is a prerequisite for further conformational changes that lead to exposure of the fusion loop. In addition, previous studies have demonstrated the pivotal role of acidic pH and the presence of Ca^2+^ in facilitating GP’s structural transformation, leading to the exposure of the fusion loop, membrane attachment, and lipid mixing.^11,17−20^ Hence, we examined how alterations in environmental conditions impact the protein–membrane interaction.

In this work, we utilized two innovative methods to probe the dynamics and mechanics of primed GP–membrane interactions. These methods only probe the protein–membrane binding step and do not address fusion. First, we developed a single-particle tracking experiment that allows multiplexed measurements, revealing that primed GP associates with membranes in the absence of the NPC1 receptor. Then, a single-molecule approach utilizing high-resolution optical tweezers was employed to measure the GP–membrane dissociation force. Our findings demonstrate that acidic pH significantly amplified GP–membrane interactions. Intriguingly, acidic pH did not necessarily alter the binding force itself under our experimental conditions. We show that at the optimal acidic pH, in the presence of Ca^2+^ ions, the unbinding force between the soluble GP ectodomain (GPΔTM) and the membrane is ≈55 pN or higher. Together, these data provide novel insights into the membrane association of the EBOV GP. Moreover, the methodologies presented here are versatile, offering broad applicability to various protein–membrane interaction systems.

## Results

### Single-Particle Tracking Experiments Unravel the NPC1-Independent Membrane Binding of GPΔTM

**Figure 1:**
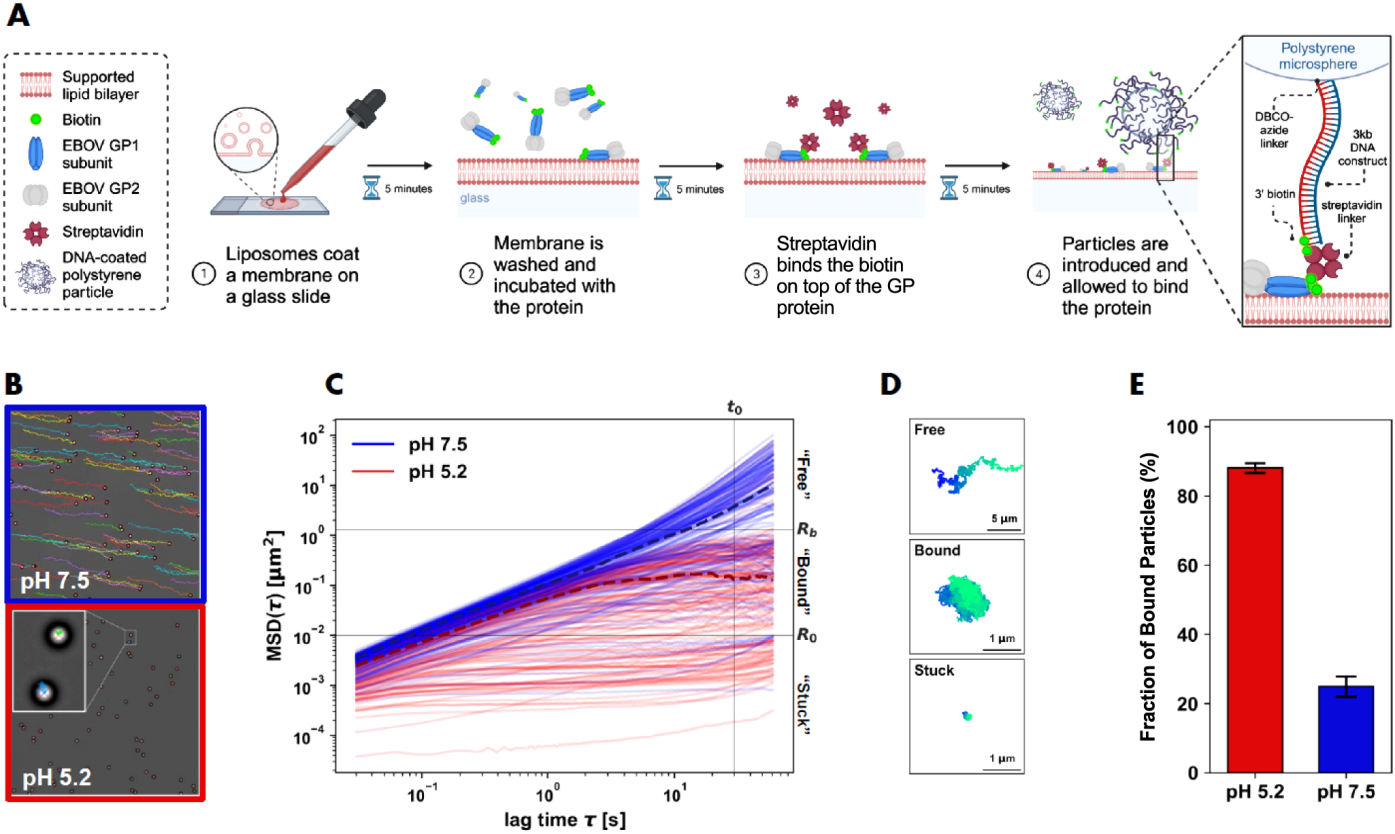
Single-particle tracking assay. (A) Schematic illustration of the sample preparation process. (1) A glass surface was coated with a synthetic membrane. (2) GPΔTM (blue and gray) labeled with biotin (green circle) was added and followed by an incubation with streptavidin (dark red) (3). (4) Particles coated with biotinylated dsDNA were then introduced and allowed to diffuse and bind the proteins. This figure was created using BioRender.com. (B) Typical trajectories of microsphere diffusion at pH 5.2 (red) and at pH 7.5 (blue). (C) The time-averaged MSD for each microsphere at pH 5.2 (*n* = 831, red) and at pH 7.5 (*n* = 822, blue). Dashed lines represent the MSD of the median particle (determined at *t*_0_ = 30 s). MSD values at *t*_0_ were used to differentiate microsphere motion (“Free,” “Bound,” “Stuck”) according to parameters *R*_b_, *R*_0_. The exponential increase of the MSDs of particles in the free and stuck regimes toward larger times is a result of the small drift affecting the solution and the membrane, respectively. (D) A representative trajectory of microsphere diffusion for each motion group. Colors represent time. (E) The percentage of “Bound” particles (excluding “Stuck”) under the examined conditions (*n* = 12 experiments each). Error bars represent the standard error of the mean (SEM). The statistical significance of the difference between the conditions, calculated by the nonparametric Mann–Whitney test, was *p* < 0.001. Data in (C) and (E) includes measurements at the indicated pH levels, both with and without Ca^2+^ ions, as Ca^2+^ presence did not result in any discernible differences.

To readily probe the interactions of GPΔTM (see the Materials and Methods section) with membranes under different conditions, we developed a high-throughput single-particle tracking assay based on bright-field video microscopy. First, a glass-supported synthetic membrane was incubated in the presence of purified, biotinylated-GPΔTM (Figure S1) and streptavidin proteins. Next, microspheres coated with biotin-labeled double-stranded DNA (dsDNA) were allowed to diffuse over the surface and bind to proteins associated with the membrane if present (Figure 1A). This was done in the presence of weak flow (Figure 1B). If the microspheres were observed to flow with the carrying liquid, then no protein–membrane association occurred. However, a restricted motion of the microspheres signified protein–membrane binding. The restriction was expected to increase with the increase in the fraction of membrane-associated proteins.

To methodically differentiate between confined and free microspheres, the time averaged mean squared displacement (MSD) of each microsphere was calculated (Figure 1C). Although freely diffusing microspheres (Figure 1B blue) exhibit a monotonic increase in their MSD with lag time, the diffusion of tethered microspheres was restricted (Figure 1B red), causing their MSD to plateau beyond a certain lag time. We selected a lag time *t*_0_ = 30 s after which the confined microspheres’ MSDs had reached a plateau. Subsequently, we defined two critical parameters to categorize microspheres into three distinct groups based on their motion: the minimum movement radius *R*_0_ = 0.01 μm^2^, used to filter out the nonspecifically stuck particles (for which MSD(*t*_0_) < *R*_0_), and the confinement radius *R*_b_ = 1.3 μm^2^, used to distinguish between free (MSD(*t*_0_) > *R*_b_) and bound (*R*_0_ < MSD(*t*_0_) < *R*_b_) particles (see Figure S2, horizontal lines in Figure 1C, D). The particle binding probability was then quantified as the fraction of bound microspheres (Figure 1E). We note that *R*_0_ and *R*_b_ depend on the experimental procedure, including membrane composition and the agitation of the surface, which affect the particle attachment to the surface (see Figures S2 and S3).

In control experiments conducted in the absence of GPΔTM, the percentage of bound particles was significantly smaller than that in the presence of the protein. Additionally, in experiments conducted in the absence of GPΔTM and on a membrane incorporating biotinylated lipids, the percentage of bound particles was close to 100% (Figure S3).

**Figure 2:**
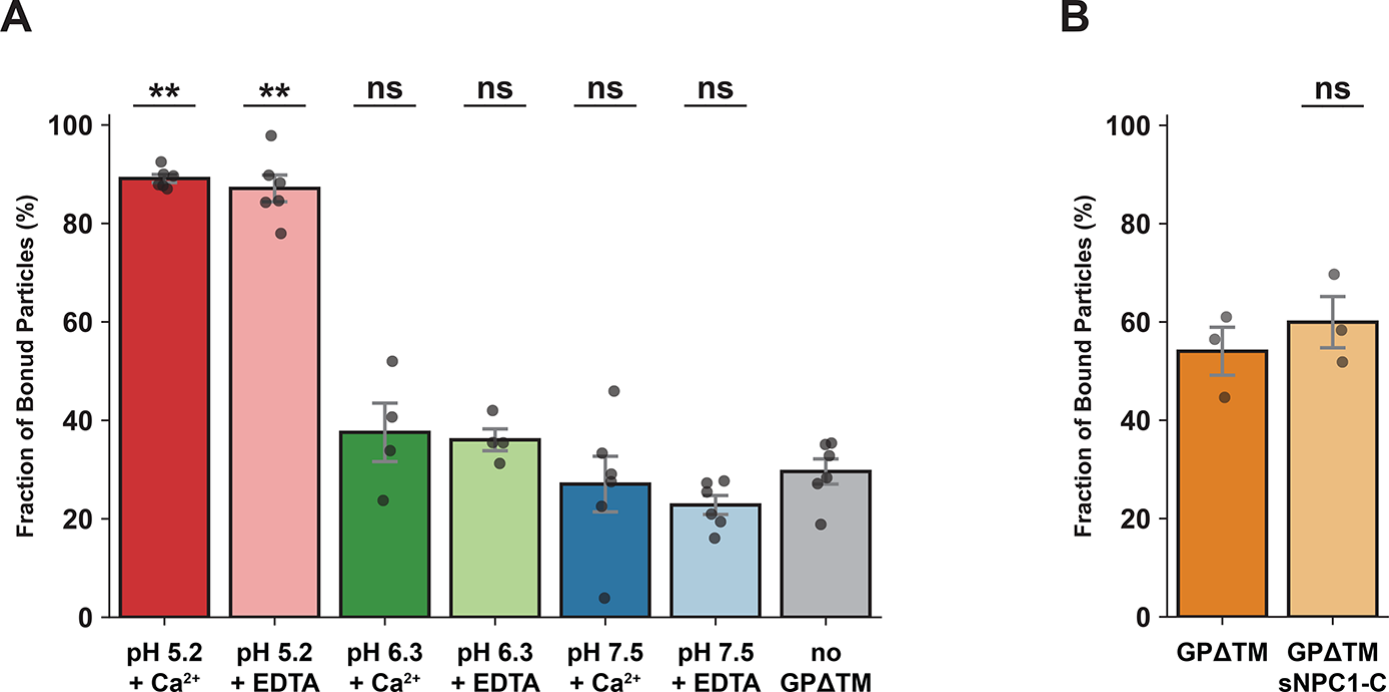
(A) The fraction of bound particles under different pH conditions (pH 5.2, 6.3, 7.5) in the presence of Ca^2+^ ions or EDTA. Statistical significance was calculated in relation to experiments done in the absence of GPΔTM. (B) The fraction of bound particles in the presence and absence of sNPC1-C, measured at pH 5.2 in the presence of Ca^2+^. The difference between the left column in (B) and the leftmost column in (A) stems from the difference in protein concentration and incubation time (see the Materials and Methods section). Error bars represent the standard error of the mean (SEM). The statistical significance for all plots was determined by the nonparametric Mann–Whitney test (* being *p* < 0.05, ** being *p* < 0.01, and ns being nonsignificantly different). All results described in this figure, including mean values, SEM values, and the number of measurements and particles are available in Table S1.

We have used our method to study GPΔTM–membrane interactions at three different pH conditions in the presence of Ca^2+^ or EDTA. We have also studied the effects of the soluble luminal domain C of the NPC1 receptor (sNPC1-C) on GPΔTM–membrane binding. Strikingly, at acidic pH, the majority of microspheres exhibited restricted diffusion, as evidenced by a mean fraction of bound particles of 89.12 ± 0.83% and 87.12 ± 2.71% in the presence of Ca^2+^ ions and EDTA, respectively (Figure 2A). Conversely, at pH 7.5, most particles diffused freely, leading to a mean of 27.06 ± 5.65% bound particles in the presence of Ca^2+^ and 22.81 ± 1.92% in the presence of EDTA (Figure 2A). In all pH conditions, no significant difference was observed in binding in the presence or absence of Ca^2+^ ions, in line with previous work with similar lipid composition.^20^ Interestingly, combining all data (regardless of the presence of Ca^2+^ ions), a significant difference (*p* < 0.01) is observed between the three tested pH conditions.

To study the effect of the NPC1 receptor on GP–membrane interaction, we conducted experiments involving GPΔTM preincubated with sNPC1-C, at pH 5.2 and in the presence of Ca^2+^ ions (as these exhibit the highest fraction of bound particles of all tested conditions). No significant difference was observed, indicating that the binding of sNPC1-C to primed GPΔTM does not enhance the interaction between GPΔTM and the membranes. Together, these findings show that GPΔTM associates with the membrane even in the absence of the NPC1 receptor, favorably binding in acidic pH.

### Measuring Single-Molecule GPΔTM–Membrane Interactions

**Figure 3:**
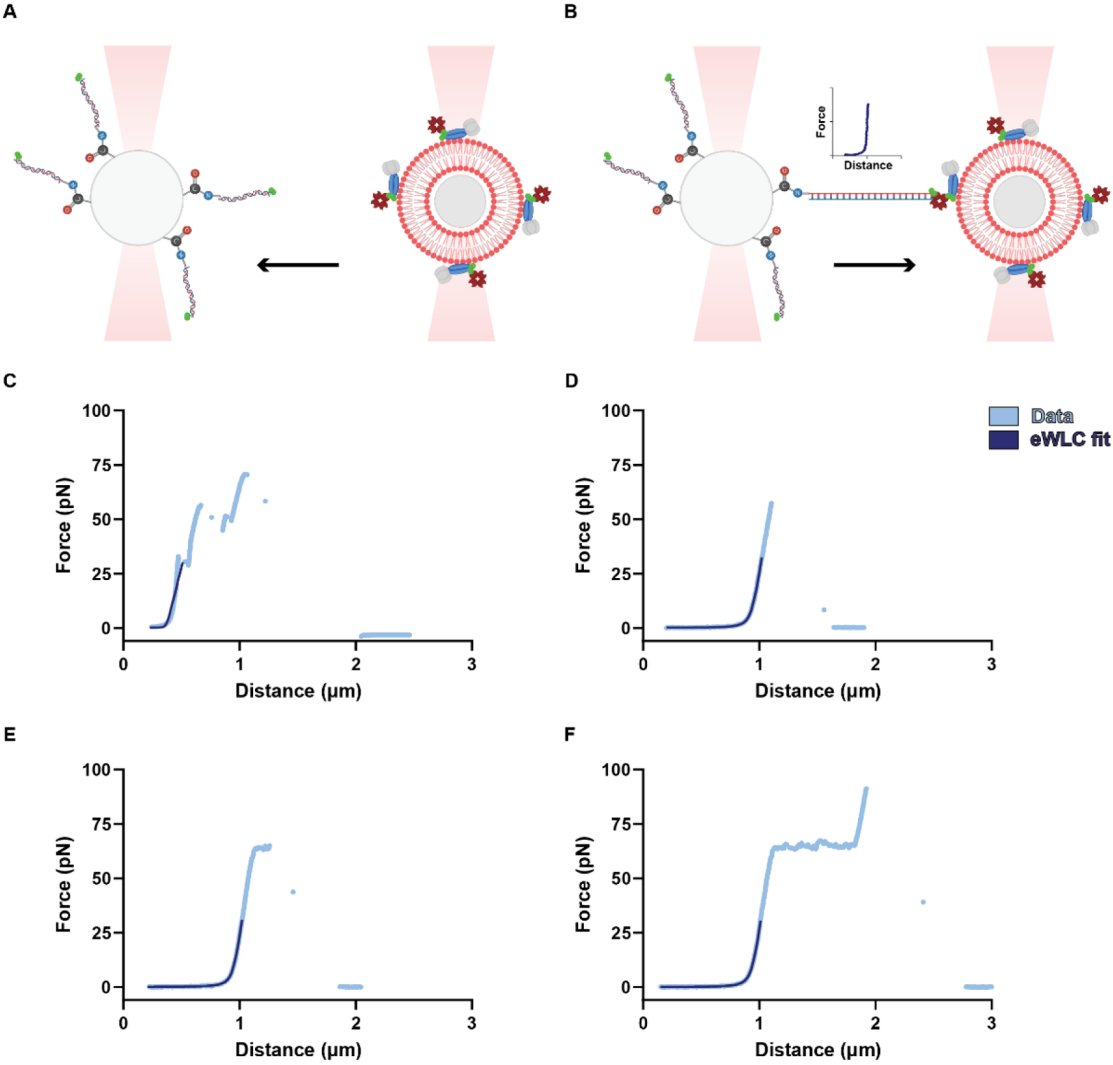
Single-molecule protein unbinding measurements. (A, B) Schematic illustration of the single molecule approach for measuring the interactions between GPΔTM and the membrane. This figure was created using BioRender.com. (A) A carboxyl microsphere (left) covalently coated with dsDNA molecules labeled with biotins (green circles) was captured by one of the traps, while the membrane-coated microsphere (right), harboring biotinylated GPΔTM (blue, gray, and green)–streptavidin (dark red) complex, was captured by the second trap. (B) The traps were brought into close proximity and then separated, exhibiting an FD relationship characteristic of an interaction event. (C–F) Representative plots of experimental interactions: multimolecular interaction (C), single-molecule interactions with a disassociation occurring before/during/after the OS stage (D, E, F, respectively).

To measure the force needed to dissociate a single GPΔTM protein from the membrane, we used a combination of optical tweezers and microfluidics. We employed a specialized experimental setup using two polystyrene microspheres (Figure 3A,B). One of the microspheres featured a covalently bound 3-kilobase-pair (3 kb) dsDNA molecule, tethered via an amide bond, while the second contained a membrane-associated GPΔTM–streptavidin complex (Figure 3A). The experimental procedure involved the initial trapping of both microspheres and their subsequent close approach and separation. In the event of an interaction between dsDNA and the GPΔTM–streptavidin complex associated with the membrane, the dsDNA molecule underwent mechanical stretching. This stretching resulted in the measurement of a well-defined force–distance (FD) relationship^21,22^ (Figure 3B).

Figures 3C–F and S4 show representative FD plots illustrating the observed classes of experimental interactions: (i) an FD curve without any force changes, indicating that there was no binding event between the dsDNA molecule and streptavidin–biotinylated GPΔTM complex (Figure S4). (ii) Stretching events corresponding to multiple dsDNA molecules that deviate from the extensible Worm-Like Chain (eWLC) model^23^ (Figure 3C). These interactions were excluded from consideration when determining the forces of unbinding events. (iii) FD plots corresponding to single-molecule dsDNA stretching events, facilitated by a single-molecule interaction occurring between GPΔTM and the membrane (Figure 3D–F). These plots were fitted to the eWLC model^23^ and the plots exhibiting the best fit parameters (see Materials and Methods section) were further analyzed to determine the magnitude of the unbinding force. Those plots can be further classified into three distinct groups based on the forces observed at the breaking point: First, plots with breaking-point forces below ≈60 pN, where detachment occurs prior to the overstretching (OS) transition (Figure 3D). Second, those exhibiting forces at ≈65 pN, featuring detachment during the OS transition (Figure 3E). Finally, plots representing the highest break forces, which entail an additional extension stage following the OS transition (Figure 3F). Together with the single-particle tracking results, these data demonstrate that GPΔTM exhibits direct interaction with the lipid membrane, independent of receptor binding.

### GPΔTM Strongly Associates with the Membrane

**Figure 4:**
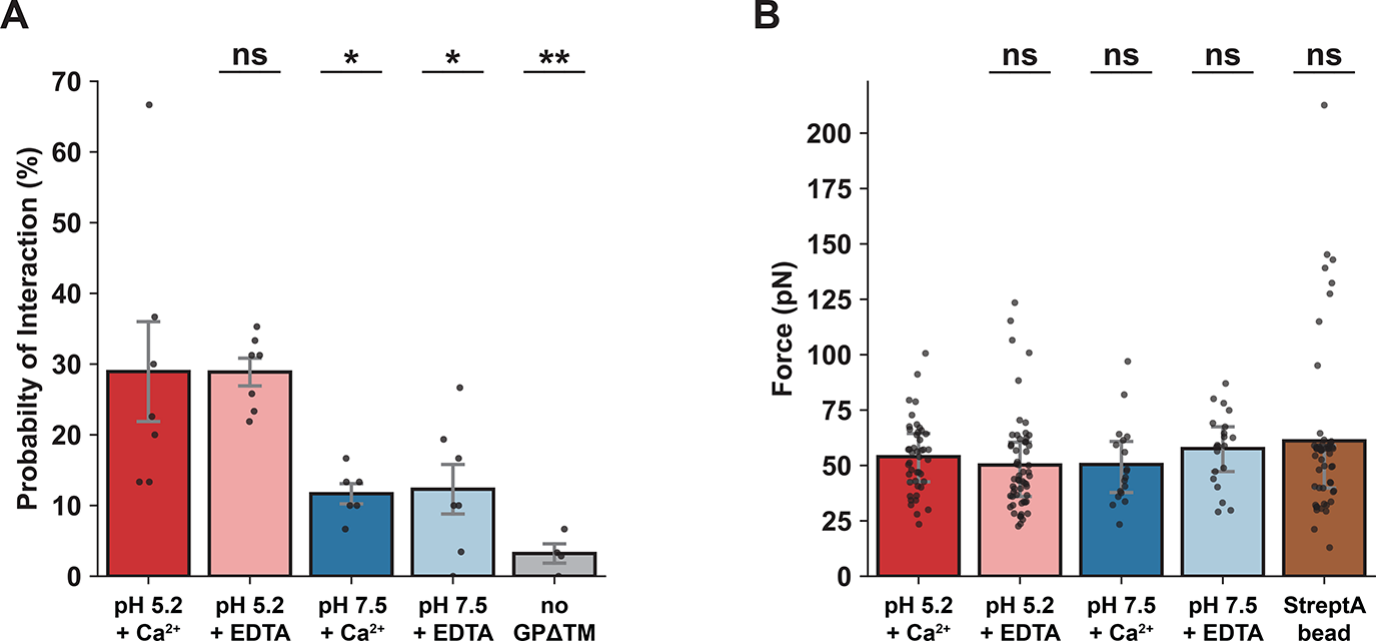
Probability of interactions and analysis of single-molecule unbinding forces at different experimental conditions. (A) The probability of interactions between the biotinylated GPΔTM–streptavidin complex and the biotinylated dsDNA molecules at pH 5.2 in the presence of Ca^2+^ (*n* = 7) or EDTA (*n* = 7), pH 7.5 in the presence of Ca^2+^ (*n* = 6) or EDTA (*n* = 7), and pH 5.2 in the presence of Ca^2+^ but without GPΔTM (*n* = 4). Each trial (black dot) consisted of at least 10 different pairs of beads and 3 approach-separation cycles for each. Error bars (gray) represent SEM. (B) Bar plots show the mean rupture forces corresponding to the various experimental conditions. StreptA bead (brown) symbolizes the experiments performed with covalently coated streptavidin microspheres at pH 5.2 in the presence of Ca^2+^. Error bars (gray) represent IQR, while each black dot is a single-molecule interaction. The statistical significance for all plots was determined by the nonparametric Mann–Whitney test (* being *p* < 0.05, ** being *p* < 0.01, and ns being nonsignificantly different).

As previously described, to assess the specificity of our experimental setup, we sought to measure the probability of interactions between GPΔTM and the membrane under varying environmental conditions (Figure 4A). Following the single-particle tracking measurements, we decided to focus our efforts on the extreme conditions of protein binding, measuring at pH 5.2 and 7.5. Consistently with the single-particle tracking, we observed the highest probability of interactions at acidic pH in the presence of Ca^2+^, with a mean value of 28.9 ± 7.1%, and in the presence of EDTA, with a mean value of 28.9 ± 2.0%. Furthermore, at pH 7.5, in the absence or presence of Ca^2+^ ions, the percentage of interactions was significantly lower (*p* < 0.05) compared to acidic conditions, with mean values of 12.3 ± 3.5% and 11.7 ± 1.4%, respectively (Figure 4A). Importantly, in the absence of GPΔTM protein, the probability of interactions was nearly negligible, measuring at 3.2 ± 1.4% (Figure 4A). Therefore, these results strongly suggest that the observed dsDNA stretching (Figure 3C–F) correlates with GPΔTM’s membrane association.

Next, we sought to elucidate the impact of acidic pH and the presence of Ca^2+^ on the dissociation force of GPΔTM and the membrane. For this purpose, we exclusively analyzed FD curves representing single-molecule interactions (Figure 3D–F). The mean rupture force in the presence of GPΔTM and Ca^2+^, at pH 5.2 was 54.0 pN (IQR = 42.6–64.4 pN). In the absence of Ca^2+^, the mean rupture force was 50.2 pN (IQR = 36.0–60.5 pN) (Figure 3B). Intriguingly, the rupture force at pH 7.5 in the presence or absence of Ca^2+^ exhibited negligible variation (*p* > 0.05), yielding mean values of 50.4 pN (IQR = 37.8–60.9 pN) and 57.6 pN (IQR = 47.3–67.5 pN), respectively (Figure 3B).

These results prompted us to consider the following possible scenarios: first, in this assay, we were using a recombinant and “primed” protein (with its glycan cap removed). Hence, some GPΔTM molecules, although at a significantly lower probability (Figure 4A), may associate with the membrane, regardless of the experimental conditions. Importantly, similar results were reported previously concerning the ability of GPΔTM to induce hemifusion of membranes.^11^ Second, the rupture events could arise from the detachment of biotin and streptavidin molecules. Our experimental setup entails a complex system comprising a biotinylated dsDNA molecule, a biotinylated GPΔTM anchored to the membrane, and the streptavidin protein serving as a linker between them (Figure 3A,B). Consequently, ruptures may occur during GPΔTM disassociation from the membrane or when biotins located on the dsDNA or GPΔTM, uncouple from streptavidin (it is important to note that covalent bonds sustain considerably higher forces^24^).Therefore, we conducted control measurements of rupture forces between biotinylated dsDNA and a covalently coated streptavidin microsphere. Here, we measured a mean force of 61.1 pN (IQR = 39.8–59.0 pN), with no significant difference in the magnitude of the forces compared to GPΔTM (*p* > 0.05) (Figure 4B). Therefore, we conclude that under our experimental conditions, at the optimal acidic pH, in the presence of Ca^2+^ ions, the unbinding force between GPΔTM and the membrane is ≈55 pN or higher. These results do not allow us to conclude whether the binding forces remain constant across all conditions. As a result, we are reporting the lower bound of the force values.

### Dynamic Force Spectroscopy Revealed Loading Rate Dependency of the Rupture Forces

As previously discussed, the rupture between the microspheres is expected to occur at the weakest point, which is anticipated to reside at the interface between GPΔTM and the membrane or the biotin–streptavidin complex. As an additional validation of our results, we employed dynamic force spectroscopy.^25−27^ This approach explores how the rate of force application affects the unbinding pathways and the intrinsic molecular bond lifetime of biological molecules such as proteins or protein complexes.

**Figure 5:**
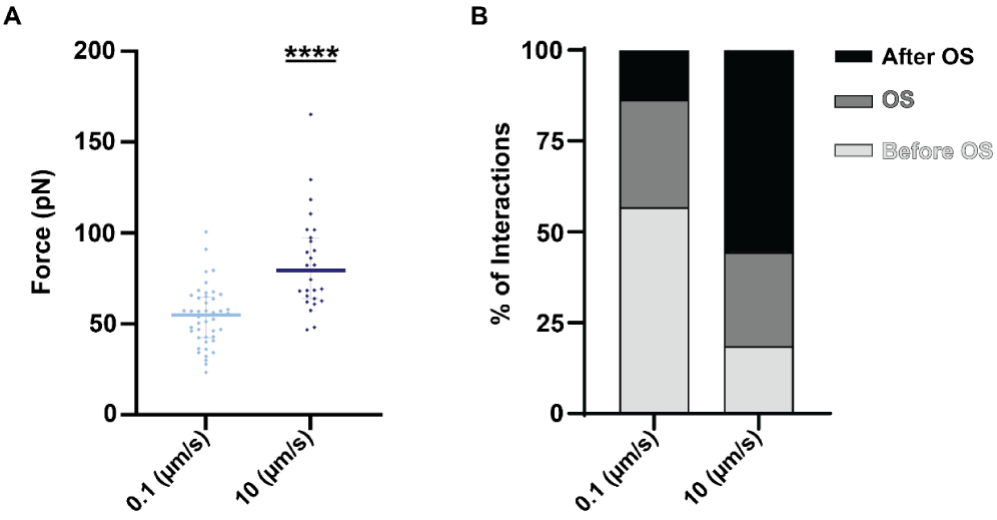
Analysis of single-molecule interactions using the dynamic force spectroscopy approach. (A) The single-molecule median rupture forces at loading rates of 16.6 pN/s (0.1 μm/s) and 1658 pN/s (10 μm/s). Gray bars represent IQR, while each dot is a single-molecule interaction. The statistical significance was determined by the nonparametric Mann–Whitney test (**** being *p* < 0.0001). (B) The percentage of single-molecule interactions with disassociations before (light gray), during (gray), and after (black) the OS phase.

We conducted our experiment at low and fast separation velocities, 0.1 μm/s (16.6 pN/s) and 10 μm/s (1658 pN/s), respectively (Figure 5A). The median rupture force measured at 0.1 μm/s was 54.9 pN (IQR = 42.5–64.8 pN), whereas at 10 μm/s, it was significantly higher (*p* < 0.05), with a median value of 79.4 pN (IQR = 64–97.5 pN) (Figure 5A). Similar unbinding forces were measured in previous studies for streptavidin–biotin interactions.^28,29^ There are multiple pathways for rupture. At lower loading rates, corresponding to slower separation velocity, the system has enough time to relax to lower-energy intermediate configurations during its transition through the rupture pathway. Consequently, the energy barrier for rupture is lower and occurs spontaneously at a weaker force. In contrast, at fast pulling rates, the system has no time to relax to the lowest-energy pathway and the needed force for spontaneous rupture increases. This phenomenon is well documented in previous works.^25−28^

To better illustrate the effect of the loading rate on the unbinding force, we analyzed and categorized the rupture events by the stage at which they occurred (Figure 5B). While pulling at 0.1 μm/s, the majority (56.8%) of the single-molecule breaking events were observed prior to the OS transition, with only a small fraction (13.6%) occurring after this stage (Figure 5B). In contrast, at 10 μm/s, most rupture events occurred after the OS stage (55.6%), while only a minority (18.5%) occurred before it (Figure 5B). The proportion of rupture events taking place during the OS remained relatively consistent (29.5% at 0.1 μm/s and 25.9% at 10 μm/s; Figure 5B). Collectively, our results demonstrate that the rupture predominantly occurs during the detachment of one of the protein complexes, rather than being attributed to any technical limitations of the experiment.

## Discussion

EBOV is responsible for one of the deadliest epidemic diseases. Hence, a better understanding of the viral pathological mechanisms is vital. In this study, we introduced two innovative methods to probe the nature of the EBOV GP membrane interaction. Our single-particle tracking experimental approach demonstrated GPΔTM’s interaction with the membrane, even in the absence of its endosomal receptor. Intriguingly, our single-molecule assay, employing optical tweezers, not only confirmed the specificity of this interaction but also underscored its remarkable strength.

NPC1 is essential for EBOV entry,^9−30^ although its mechanistic role in promoting fusion is unclear. Our results demonstrate that NPC1 binding is not a prerequisite for the membrane association of primed GP (Figures 2 and 4A). Remarkably, our data demonstrate a robust association comparable in strength to the bond between streptavidin and biotin (Figure 4B). Therefore, we speculate that the key factors promoting membrane association are the conditions within the endosome (acidic pH and the presence of Ca^2+^). This interpretation is also consistent with single-molecule fluorescence studies, which demonstrate that acidic pH and Ca^2+^ promote a GP conformation that is competent for membrane association.^11,20^ NPC1 may serve as a coordinating factor, clustering multiple GPs to promote efficient fusion. Alternatively, interaction with full-length membranous NPC1 may increase the likelihood that pH-mediated GP conformational changes lead to productive engagement with the membrane. We intend to explore the role of NPC1 upstream of the membrane binding step in future studies. Importantly, similar findings have been reported for other class I fusogens. The ACE1 receptor is not biochemically required for the fusion process mediated by the SARS-CoV2 Spike protein.^31^ Similarly, the LAMP1 receptor is not required for Lassa virus fusion, but raises the pH at which fusion occurs.^32−34^

A lingering question remains regarding the nature of the interaction we measured. One possibility involves membrane docking or just a shallow penetration by the fusion loop, akin to the behavior of Synaptotagmin-1, a Ca^2+^ sensor protein crucial for neurotransmitter release.^35^ Upon cation binding, Synaptotagmin’s soluble domains penetrate the membrane shallowly.^36^ Further investigation, using optical tweezers, has shown that the membrane unbinding force of these domains was relatively low, 2–7 pN.^37^ The second option suggests a deeper binding mode, as demonstrated previously for the SARS-CoV2 Spike protein fusion loop.^38^ Here, subsequent atomic force microscopy (AFM) experiments revealed that the strength of the disassociation of the protein’s fusion loop from a hydrophobic surface was 1.91 nN,^39^ substantially higher than for Synaptotagmin-1–membrane disassociation. In addition, a recent computational study has shown that class I fusogens have the highest membrane-binding affinities, rationalized by the deep insertion of their hydrophobic fusion loops.^40^ Given the disassociation forces measured by us (Figure 3B) were within the range of dozens or hundreds of pN, we hypothesize that the fusion loop of GP inserts deeply into the membrane, allowing for the interaction with the membrane’s hydrophobic core. Further single-molecule experiments with DNA or another linker, using AFM and heterobifunctional covalent bonds, would reveal the absolute strength and affinity of GP’s membrane association. We further note that we cannot exclude a stronger association with the membrane in the presence of NPC1 based on our results.

Based on our unbinding forces results, we aimed to estimate the lower bound of the unbinding energy of the GPΔTM. The minimal unbinding force is roughly 55 pN. Assuming that GPΔTM−membrane unbinding force is similar to or higher than that of streptavidin−biotin, the unbinding energy is given by the integral of the pulling force over the normal direction to the membrane, , with *z*_0_ as the peptide center of mass insertion depth, *F*_*z*_ as the force applied to the fusion peptide, and  being the normal direction. It is challenging to calculate Δ*G* based on the force–displacement curve obtained using the optical tweezers setting (Figure 3C–E) since most of the displacement is due to the stretching of the dsDNA (μm scale), while the maximal displacement of the fusion peptide before it unbinds from the membrane cannot be larger than membrane thickness, 3–4 nm. Therefore, we estimate the Δ*G* by considering *F*_*z*_ as the maximal rupture force, which we assume to be constant during the pulling of the fusion peptide from the membrane. We take *z*_0_ to be in the range of 0.8–1.2 nm based on MD simulations.^41^ Based on this rough estimation, we find the unbinding energy to be 13 *k*_B_*T* (IQR = 8–19 *k*_B_*T*). This estimation agrees with previous works that found Ebola GP fusion peptide binding energy to be 12 *k*_B_*T* both experimentally^17^ and computationally.^41^ Interestingly, the binding energies of Hemagglutinin fusion peptide of the Influenza virus, which also fuses within the late endosome,^42^ are similar in magnitude and are also strengthened in lower pH with binding energies of 12.6 *k*_B_*T* at pH 7 and 14 *k*_B_*T* at pH 5.^43^ Moreover, HIV and SARS-CoV2 fusion peptides also have comparable binding energies to membranes, 14 *k*_B_*T*(44) and 14–16 *k*_B_*T*,^45^ respectively.

Working with a recombinant, primed protein, excluding all other cellular components, as well as using synthetic membrane mimetics may be very different from the physiological scenario. We note that the extent of reversible sampling of prefusion conformations by GP might differ between the full-length protein and its ectodomain. Previous experiments comparing the prefusion conformational dynamics of Ebola GP^46^ demonstrated that the dynamics of GP on virus particles were higher than that on recombinant trimer. In the current study, we used a cap-cleaved GP, which might further affect the protein dynamics. As the complexity of the full biological system is very large, investigating a well-controlled, simple model system can significantly enhance our understanding of the roles of each component. We thus believe that our results offer valuable insights by challenging existing paradigms and providing a foundation for further exploration.

## Materials and Methods

### GPΔTM Plasmid Preparation

EBOV glycoprotein (GenBank: KJ660346.2) lacking the mucin-like domain and containing an additional adenosine at nucleotide 890^47^ was modified to remove amino acid residues 633 to 676 of the transmembrane/intracellular domains and add a C-terminal trimerization domain and 6x His Tag. The gene was inserted as a gBlock (Genescript) into the pHL-sec mammalian expression vector (Addgene #99845). A WELQut Protease cleavage site was added by replacing amino acids 191 to 194 (amino acids KDFF in the native sequence) with the amino acids WELQ by site-directed mutagenesis. To generate the final EBOV-Mak-GPΔTM-WELQ194-BAP expression construct, a BirA biotin ligase acceptor peptide (BAP) sequence^48^ was introduced between the trimerization domain and 6× His Tag by overlap extension PCR using the following primers (Integrated DNA Technologies, Inc.):

Forward:

5′-TCGAGGCCCAGAAGATCGAGTGGCACGAGGGCTCTGGCCACCACCATCAC-3′

Reverse:

5′-CTGGGCCTCGAAGATGTCGTTCAGGCCAGAGCCCTTGGTACCCAGAAATG-3′

### Protein Expression and Purification

EBOV-Mak-GPΔTM-WELQ194-BAP (GPΔTM) was produced in Expi293F cells (Thermo Fisher) according to the manufacturer’s instructions. Biotin was present in the cell culture media; therefore, the BirA enzyme could ligate free biotin to the BAP on the GP C-terminus. Plasmids encoding EBOV-Mak-GPΔTM-WELQ194-BAP, Furin protease, and secreted BirA-Flag (Addgene no. 64395) were cotransfected at a ratio of 6:1:0.75 using the ExpiFectamine 293 Transfection Kit (Thermo Fisher). At 5 days post-transfection, the cell culture supernatant was harvested and purified using Ni-NTA Agarose (Invitrogen). The protein was exchanged into phosphate-buffered saline (PBS) using a Vivaspin 6 ultracentrifugation spin columns (Sartorius). The sample was cleaved with WELQut protease (Thermo Fisher) to remove the glycan cap by incubation with one unit WELQut protease per 25 μg of protein at 30 °C for 16 h. The primed (i.e., glycan cap-cleaved) protein was further purified via size exclusion chromatography on a Superdex 200 Increase 10/300 GL column (GE Healthcare) in PBS. Peak fractions were evaluated by sodium dodecyl sulfate-polyacrylamide gel electrophoresis (SDS-PAGE) and by immunoblot with the anti-GP1 monoclonal antibody HC38 to detect the cap-cleaved GP, as previously described,^46^ or Precision Protein StrepTactin-HRP Conjugate (BioRad) to detect biotin. Protein containing fractions were stored at −80 °C.

The sNPC1-C^49^ was expressed in Expi293F cells (Thermo Fisher) using the ExpiFectamine 293 Transfection Kit (Thermo Fisher). At 72 h post-transfection, cell culture supernatant was harvested and purified following an overnight incubation with Ni-NTA Agarose (Invitrogen). The protein was exchanged into phosphate-buffered saline (PBS) and concentrated using Amicon Ultra Centrifugation Filters (Millipore Sigma), before storage at −80 °C. Protein integrity was assessed by SDS-PAGE before use.

### 3 kb Sequence Plasmid DNA Preparation

A commercially available short biotinylated and digoxigenin (DIG) labeled 3 kb DNA (Lumicks) was subcloned using 5′XhoI and 3′*Eco*RI (NEB) into the pEGFP-N1 vector (Addgene #6085-1). The primers (IDT) used to amplify the DNA sequence by PCR were:

Forward:

5′-AAACTCGAGGGCAGGTGAAGGACTCCTTCGGC-3′,

Reverse:

5′-AAAAAGAATTCCAGTTCGCTGCACTGCTCAATGCG-3′.

After employing the restriction enzyme-based cloning technique, the construct was transformed into chemically competent DH5α *Escherichia coli* cells (Thermo Fisher). The success of the procedure was determined by Sanger sequencing (ZABAM Instrumentation and Service sequencing unit at Tel Aviv University), and this construct was used as a PCR template for further DNA amplifications.

### Hetero-Bifunctional 3 kb dsDNA Construct Preparation

First, to generate the 5′-end labeling of the 3 kb dsDNA’s leading strand with a dibenzocyclooctyne (DBCO) or a primary amino group, we used the following modified primers (IDT):

Forward:

5′-5DBCON/GGCAGGTGAAGGACTCCTTCGGCGGGATGAT-3′

or:

5′-5AmMC6/GGCAGGTGAAGGACTCCTTCGGCGGGATGAT-3′,

respectively. The reverse primer, containing the *Eco*RI restriction site, was the same as that used in the previous section. The reaction was carried out using a labcycler (SensoQuest), with an annealing temperature of 65 °C, using the Phusion High-Fidelity PCR Master Mix X2 (NEB), followed by the residual methylated template DNA digestion by DpnI (NEB) at 37 °C for 1 h. Subsequently, the product was loaded onto a 1% Agarose (Grisp) gel containing the SYBR Safe DNA Gel Stain (Thermo Fisher) and followed by gel electrophoresis (Benchmark) for 20 min at 100 V. The dsDNA band, corresponding to the correct molecular weight was excised from the gel and purified using NucleoSpin Gel and PCR Clean-up (Macherey-Nagel) according to the manufacturer’s instructions. To generate the 3′-end labeling of the leading strand, the purified product was further subjected to a restriction reaction by *Eco*RI (NEB) at 37 °C for 1 h. Next, the restricted product was purified again using the same purification kit, and Klenow (NEB, M0210) end-filling was performed in NEBuffer 2 (NEB) for 15 min at 25 °C. Besides the restricted dsDNA and Klenow, this reaction (50 μL in total) contained 100 μM of each: dATP, dCTP, dGTP, and biotinylated dUTP (Jena Bioscience). To remove the buffer components and nucleotides, the final product was purified again using the cleanup kit. Finally, the heterobifunctional dsDNA concentration (in ng/μL) was determined photometrically at a wavelength of 260 nm by NanoDrop One (Thermo-Fisher Scientific) and converted to pmol using the https://worldwide.promega.com/resources/tools/biomath Web site.

### Small Unilamellar Vesicles (SUVs) Preparation

For the single-particle tracking experiments, the following molar ratios, 74.5% of 1,2-dioleoyl-*sn*-glycero-3-phosphocholine (DOPC, 350 μg) (Anatrace), 5% 1,2-dioleoyl-*sn*-glycero-3-phosphatidylserine (DOPS) (Anatrace), 20% of cholesterol (Sigma-Aldrich), and 0.5% of 1,2-dioleoyl-*sn*-glycero-3-phosphoethanolamine-N-(lissamine rhodamine B sulfonyl) (Rh-PE) (Avanti), were combined in a disposable glass vial (all dissolved in chloroform). For control experiments, the composition 79.5% DOPC, 20% cholesterol, and 0.5% Rh-PE was used. In the case of membranes containing biotin, 1% biotinyl PE (Avanti) was used instead of DOPC. For optical tweezers experiments, the composition was 74% DOPC (70 μg), 5% DOPS (Anatrace), 20% cholesterol, and 1% Rh-PE. The chloroform was dried under argon and then under vacuum for 1–2 h. Next, the lipids were rehydrated with 1 mL of rehydration buffer containing 150 mM NaCl (Carlo Erba Reagents) and 20 mM 4-(2-Hydroxyethyl)-1-Piperazineethanesulfonic Acid (HEPES), pH 7.5 (Thermo Fisher) for 10 min at room temperature (RT). For single-particle tracking, the lipids were rehydrated in the same way but with tris(hydroxymethyl)aminomethane (TRIS, Bio-Lab) buffered saline (TBS). The rehydrated lipids were further extruded (Avanti miniextruder) 13 times, using a 0.1 μm membrane (Whatman), to form homogeneous SUVs.

### Coating Microspheres with DBCO-Biotin Labeled dsDNA

Microspheres were coated with DBCO-biotin labeled dsDNA using the strain-promoted alkyne–azide cycloaddition (SPAAC) technique.^20^ 15 mL of homogeneously suspended 1% (w/v), 5.32 μm azide polystyrene microspheres (Spherotech) were supplemented with 0.3 pmol of DBCO-biotin labeled dsDNA, and the reaction volume was adjusted to 500 mL with PBS buffer. Next, the reaction tube was protected from light and incubated on a rotating device (Biosan) overnight at RT. Subsequently, the labeled beads were rinsed once with TBS by centrifugation. Finally, the supernatant was removed to obtain a final volume of 50 μL beads slurry.

### Assembly of Open-Ended Flow Chamber for Single-Particle Tracking Experiments

Silica slides (26 × 76 × 1 mm, Bar Naor) and coverslips (24 × 24 × 0.14 mm, Marienfeld-Superior) were rinsed with ethanol (Bio-Lab) and double distilled water (DDW). Subsequently, they were immersed in a 4 M potassium hydroxide solution (Rhenium) and sonicated using a bath sonicator (Elma S30H) for 90 min at RT. Next, the slides were thoroughly washed with DDW and dried using nitrogen gas. To construct an open-ended flow chamber, we carefully positioned two thin parafilm spacers along the edges of the hydroxylated silica slide. The coverslip was then gently placed atop these spacers, creating a defined space. Finally, this entire assembly was sealed by melting the parafilm spacers using a hot plate.

### Membrane and Protein Coating for Single-Particle Tracking Experiments

To remove aggregates, aliquots of GPΔTM and sNPC1-C were quickly thawed and centrifuged for 20 min at 20,000*g*. sNPC1-C was then diluted by a factor of 50 into PBS and filtered through a 0.22 um PDFV membrane (Merck). The concentration of SUVs was determined using NanoSight NS300 (Malvern Panalytical) and adjusted to ≈8 × 10^11^ particles/mL in either 100 mM sodium acetate buffer pH 5.2 (Thermo Fisher), 50 mM NaCl (ABS 5.2), 100 mM 2-(*N*-morpholino)ethanesulfonic acid (MES) buffer pH 6.3, 50 mM NaCl (MBS 6.3), or 100 mM HEPES, 50 mM NaCl (HBS 7.5), supplemented with 2 mM CaCl_2_ (Acros Organics). Subsequently, 30 μL of this solution was pipetted into the channel and allowed to incubate for 5 min at RT to form supported bilayers. The membrane-coated channel was then washed with a 5-fold volume of the experimental buffer (ABS 5.2, MBS 6.3 or HBS 7.5 supplemented with either 0.5 mM CaCl_2_ or 5 mM ethylenediaminetetraacetic acid (EDTA) (Thermo Fisher)) by applying 35 μL of buffer to one side while simultaneously draining it from the other side using a Kimwipe. Following this step, 0.8 ng/μL GPΔTM was introduced into the channel and incubated for 5 min at RT. For experiments involving the NPC1 receptor, 0.9 ng/ul GPΔTM was incubated with 3.9 ng/μl sNPC1-C for 5 min at RT, following protocols in previous work.^11^ The protein mix was then incubated on the membrane for 30 s, to mitigate nonspecific attraction of NPC1 to the membrane. The membrane was then washed 5 times, and Ca^2+^-containing buffer was switched to EDTA buffer to prevent nonspecific attachment of particles to the membrane due to Ca^2+^ bridging of DOPS lipids. Subsequently, the channel was exposed to a solution containing 1.0 ng/μL of fluorescently tagged streptavidin (Streptavidin-Dylight 633, Thermo Fisher) for an additional 5 min incubation. A thorough washing step, similar to the previous one, was performed. Then, to verify the integrity and quality of the membrane, fluorescence *z*-scans of the membrane (excitation/emission wavelengths of 546/567 nm) and streptavidin (excitation/emission 638/658 nm) were acquired (Semrock quad-band DA/FI/TR/Cy5–4X-B cube filter). Finally, the DBCO-labeled microsphere solution was diluted by a factor of 5 and applied to the channel. The channel was inverted and placed on a microscope (Nikon Eclipse Ti2-E) for further analysis.

### Single-Particle Tracking Data Acquisition and Analysis

Bright-field microscopy videos capturing the diffusion of DBCO-labeled particles were recorded at 33 frames per second rate using a 60X objective lens (NA 1.4, Nikon). To ensure sufficient binding time, each sample was incubated for 10 min. To prevent nonspecific binding and release stuck particles, the sample was gently shaken by moving the microscope stage 5 times back and forth using the joystick. Subsequently, the particles’ motion was recorded for 5 min and analyzed using the Trackpy^50^ Python implementation of the Crocker–Grier^51^ algorithm. To characterize the particle diffusion, the time-average MSD of each particle was calculated by using the following formula:
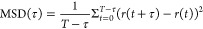


where τ is the lag time, *T* is the total length of the video, and *r*(*t*) is the position of the particle at time *t*. To avoid statistical bias generated by short trajectories of particles leaving the field of view during experiments, only trajectories longer than 1.25 min were considered for further analysis. The particles were then divided into groups based on the asymptotic value of their calculated MSD gauged at *t*_0_*=* 30 s. Finally, the number fractions of bound and free particles in each sample were used to compare the different experimental conditions.

### Microspheres Membrane, GPΔTM, and Streptavidin Coating for Optical Tweezers

The suspension for the membrane coating was prepared by rinsing 20 μL of homogeneously suspended 5% (w/v), 3.15 μm polystyrene microspheres (Spherotech), four times in total, with 450 μL of ultrapure water (twice) and rehydration buffer (twice), using centrifugation for 3 min at 900 *g* (Eppendorf) between each washing steps. Then, 400 μL of SUV mixture, supplemented with 2 mM CaCl_2_, was added to the suspension and the volume was adjusted to 500 μL with rehydration buffer. This suspension was protected from light with aluminum foil and incubated on a rotating device overnight at RT. Finally, the membrane-coated beads were washed three times with rehydration buffer using the centrifugation procedure, and the supernatant was discarded to obtain ≈30 μL of membrane-coated particle suspension.

In order to remove aggregates, an aliquot of GPΔTM was quickly thawed and centrifuged for 20 min at 20,000 *g*. Subsequently, 2 μL of the membrane-coated microspheres was incubated for 5 min at RT, with 0.03 ng/μL GPΔTM in the ABS 5.2 buffer (50 μL final) supplemented with 0.5 mM CaCl_2_ (in the absence of CaCl_2_, 1 mM EDTA was added instead). For experiments conducted at pH 7.5, the buffer was exchanged with HBS 7.5 buffer. For negative control experiments, GPΔTM was substituted with a corresponding volume of PBS. Subsequently, 10 μL of fluorescently tagged, 0.06 μg/μL streptavidin was added and incubated for 1 min at RT. Finally, the volume was adjusted to 400 μL with a corresponding assay buffer.

### Coating Microspheres with Primary Amine-Biotin Labeled dsDNA

The mixture for the primary amine-biotin labeled dsDNA was prepared using the carbodiimide cross-linking strategy.^52^ 10 μL of homogeneously suspended 5% (w/v), 3 μm carboxyl polystyrene particles (CD Bioparticles) were rinsed once with 490 μL of ultrapure water, and once with 25 mM 2-(*N*-morpholino)ethanesulfonic acid (MES), pH 5 buffer (Sigma-Aldrich). Then, *N*-(3-(dimethylamino)propyl)-*N*′-ethylcarbodiimide hydrochloride (EDC) (Sigma-Aldrich) was freshly dissolved in 25 mM MES, pH 5 and added to the prewashed carboxyl particles to a concentration of 100 mM in 100 μL of reaction volume. This mixture was incubated for 3 min at RT. Immediately after, 400 μL of 100 mM HEPES, pH 7.5, containing 0.5 pmol of the amine-biotin labeled dsDNA was added, protected from light, and incubated on a rotating device for 5 min at RT. Subsequently, the reaction was quenched (5 min, RT) with 100 mM TRIS, pH 8.5 (Bio-Lab). Finally, the coated microspheres were centrifuged for 3 min at 900 *g* and resuspended with PBS to a final volume of 250 μL. A 25-fold dilution in a corresponding buffer was used in optical tweezers experiments.

### Preparation of Streptavidin Microspheres

10 μL of homogeneously suspended 0.5% (w/v), 2.06 μm streptavidin polystyrene particles (Spherotech) were washed three times with PBS as previously described and resuspended in 1 mL of PBS.

### Optical Tweezers

The experiments were performed using a C-trap confocal fluorescence optical tweezers setup (Lumicks) as previously described.^53^ Briefly, two optical traps were used to capture polystyrene microspheres. The displacement of the trapped beads from the center of the trap was measured and converted into a force signal, while the distance was measured by piezo tracking. The trapped beads were scanned by confocal fluorescence microscopy built-in the C-trap instrument, with the excitation/emission wavelengths of 488/500–550 and 561 nm/575–625 nm, and 650–750 nm, respectively. 1% for 488 nm and 0.5% for 561 nm laser power was used for all scans when 54.35 μW is the maximal laser power.

For all interaction studies, two microsphere sets, one harboring the covalently attached biotinylated dsDNA and another containing the streptavidin-biotinylated GPΔTM–membrane complex, were used. Microspheres were injected into two different channels of a 5-channel laminar flow cell (Lumicks) and captured in two separate optical traps (Trap 1 and Trap 2). The remaining channels were flushed with the corresponding assay buffer.

Once trapped, the two microspheres were moved into buffer-containing channel 2 and imaged using the confocal fluorescence scanning microscope. This allowed differentiation between the bead types as only the membrane-coated bead emitted fluorescence. When streptavidin beads were used, differentiation between the two bead types was based on the diameter of the microspheres. Subsequently, the traps were calibrated using power spectral analysis and had a stiffness (*k*) average value of 0.17 pN/nm. All experiments were carried out at a constant *z* position and trapping power. Finally, the membrane-coated microsphere (Trap 1) was brought into proximity (≈ 0.2 μm) with the dsDNA-coated microsphere (Trap 2), followed by their immediate separation (at a speed of 0.1 or 10 μm/s). The loading rate was calculated by multiplying the average stiffness (*k*) by the separation velocity.

Data acquisition was carried out using the Bluelake software (Lumicks) and processed using Lumicks’ Pylake Python package. All data analyses were performed with custom-written Python scripts. The codes used for data analysis and model fittings are available at https://gitlab.com/alon.grossman119/ebola-paper. The interactions were categorized as single/multimolecule based on the shape of the FD plots (see Figure 2). An interaction was defined as a single molecule when a distinct OS plateau was detected at ≈65 pN. In cases where disassociation occurred before OS, we considered the calculated parameters of the 3 kb dsDNA: contour length (Lc) and persistence length (Lp). Specifically, if 0.8 ≤ Lc ≤ 1.2, the interaction was classified as single molecule. Moreover, if the Lc was higher/lower, 20 ≤ Lp ≤ 75 was also considered a single-molecule interaction. Following the analysis, the data were exported and presented using standard Python libraries (Matplotlib, Seaborn) or GraphPad Prism.

## Supporting Information Available

Design and evaluation of primed GPΔTM (Figure S1); determination of parameters *R*_0_ and *R*_b_ (Figure S2); MSD and binding fractions for control experiments (Figure S3); single-molecule experiment measurement (Figure S4); and single-particle tracking results for all conditions presented in Figure 2 (Table S1) (PDF)

## Abbreviations

ABS 5.2: acetate-buffer saline containing 100 mM sodium acetate buffer pH 5.2, 50 mM NaCl
AFM: atomic force microscopy
BAP: BirA biotin ligase acceptor peptide
DBCO: dibenzocyclooctyne
DDW: double-distilled water
DIG: digoxigenin
DOPC: 1,2-dioleoyl-*sn*-glycero-3-phosphocholine
DOPS: 1,2-dioleoyl-*sn*-glycero-3-phosphatidylserine
dsDNA: double-stranded DNA
EBOV: Ebola virus
EDC: *N*-(3-(dimethylamino)propyl)-*N*′-ethylcarbodiimide hydrochloride
FD: Force–distance
GP: glycoprotein
GPΔTM: GP delta transmembrane domain (EBOV-Mak-GPΔTM-WELQ194-BAP)
HBS 7.5: HEPES-buffer saline containing 100 mM HEPES buffer pH 7.5, 50 mM NaCl
HEPES,: 4-(2-hydroxyethyl)-1-piperazineethanesulfonic acid
IQR: interquartile range
kb: kilobase-pair
Lc: contour length
Lp: persistence length
MBS 6.3: MES-buffer saline containing 100 mM MES buffer pH 6.3, 50 mM NaCl
MES: 2-(*N*-morpholino)ethanesulfonic acid
MSD: mean square displacement
NPC1: Niemann–Pick C1
OS: overstretching
Rh-PE: 1,2-dioleoyl-*sn*-glycero-3-phosphoethanolamine-*N*-(lissamine rhodamine B sulfonyl)
sNPC1-C: soluble Niemann–Pick C1 loop C
SEM: standard error of the mean
SPAAC: alkyne–azide cycloaddition
SUV: small unilamellar vesicle
TBS: TRIS buffered saline
