# Supplementary material for: Ebola Virus Glycoprotein Strongly Binds to Membranes in the Absence of Receptor Engagement
Source: ACS Infect Dis. 2024 Apr 29;10(5):1590–601. doi: 10.1021/acsinfecdis.3c00622 (PMC11091876; doi:10.1021/acsinfecdis.3c00622)
Supplement: Supplementary file 1 — id3c00622_si_001.pdf [file id3c00622_si_001.pdf]

Supporting Information

Ebola Virus Glycoprotein strongly binds to membranes in the absence of receptor engagement

Alisa Vaknin[1,2,#] , Alon Grossman[1,2,#] , Natasha D. Durham[3] , Inbal Lupovitz[1,2] , Shahar Goren[1,2] , Gonen Golani[4], Yael Roichman[1,2,5] , James Munro[3,6,*] , Raya Sorkin[1,2,*]

1. School of Chemistry, Raymond & Beverly Sackler Faculty of Exact Sciences, Tel Aviv University, Tel Aviv, 6997801, Israel

2. Center for Physics and Chemistry of Living Systems, Tel Aviv University, Tel Aviv, 6997801, Israel

3. Department of Microbiology and Physiological Systems, University of Massachusetts Chan Medical School, Worcester, Massachusetts, MA 01605, United States of America

4. Department of Physics and Haifa Research Center for Theoretical Physics and Astrophysics, University of Haifa, Haifa, 3498838, Israel

5. Raymond and Beverly Sackler School of Physics & Astronomy, Tel Aviv University, Tel Aviv, 6997801, Israel

6. Department of Biochemistry and Molecular Biotechnology, University of Massachusetts Chan Medical School, Worcester, Massachusetts, MA 01605, United States of America

# These authors contributed equally

* Corresponding Authors: rsorkin@tauex.tau.ac.il, james.munro@umassmed.edu

This file contains:

5 Pages

4 Figures

1 Table

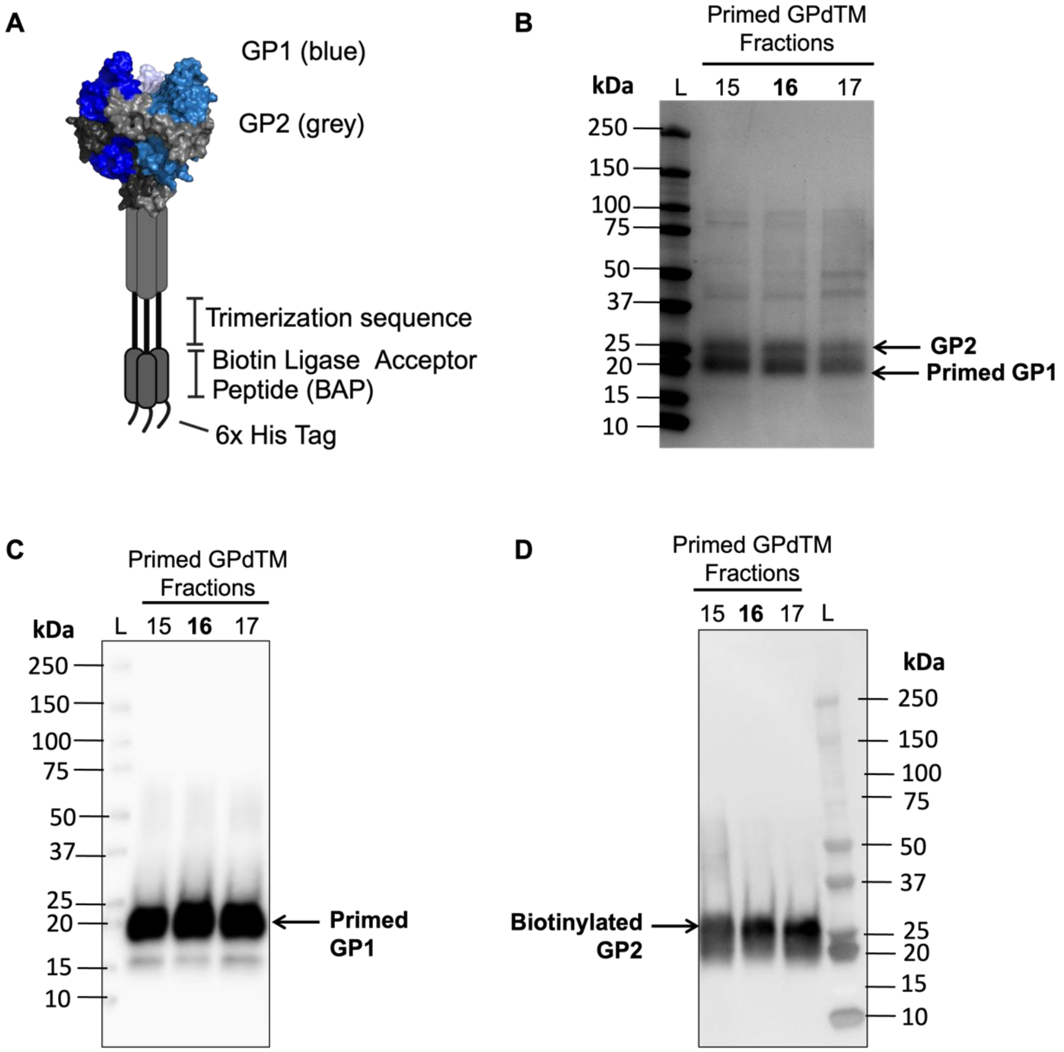

Figure S1: Design and evaluation of primed GPdTM.

A. Schematic illustration of primed GPdTM protein. Created with Biorender.com. B. SDS-PAGE of primed GPdTM after WELQut protease treatment and size exclusion chromatography. Peak fractions 15, 16, and 17 were evaluated. Fraction 16 was selected for subsequent experiments. C**.** Western blot of primed GPdTM using anti-GP1 antibody H3C8. D**.** Western blot using StrepTactin-HRP to detect biotin on GP2 of primed GPdTM.

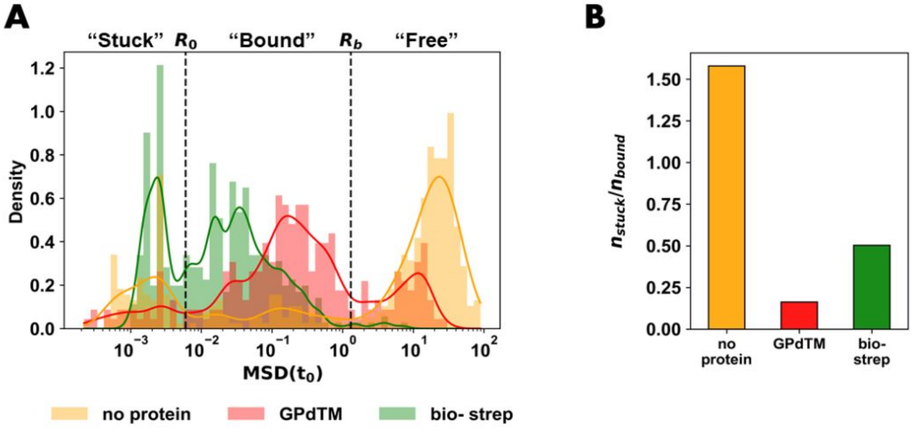

Figure S2: Determination of parameters R_0, R_b

A. A log scale histogram of the MSD values at t_0 for three experimental conditions: In the absence (yellow) and in the presence of GPdTM (red), and in the absence of GPdTM on a membrane incorporating biotinylated lipids (green, bio-strep). Bars are normalized so that the total area of each histogram equals 1. Lines represent a kernel density estimation of the distribution function. The parameters ($R_0 = 0.006\ \mu m^2$, $R_b = 1.3\ \mu m^2$) were defined on local minima of the histogram, so that the population is divided into 3 distinct groups (“Free”, “Bound”, “Stuck”). All experiments were done at pH 5.2 in the presence of Ca^{2+} ions. Stuck particles were filtered out before the statistical analysis in later figures, as they probably resulted from non-specific interactions. **B**. The ratio between stuck and bound particles in these three conditions. The higher ratio in the absence of GPdTM supports the idea that nonspecific particle confinement (“sticking”) likely results in a significant hindrance of particle motion and MSD, that is distinguishable from the tether-induced weaker confinement exhibited by the ‘bound’ particles (see Figure 1D). There is, however, a certain overlap between MSDs of non-specifically stuck particles and bound particles attached by numerous tethers, as evidenced by the higher ratio of stuck to bound particles in experiments involving membrane incorporating biotinyl-PE. In the expriments described in these figures, the surface were not agitated during particle incubation.

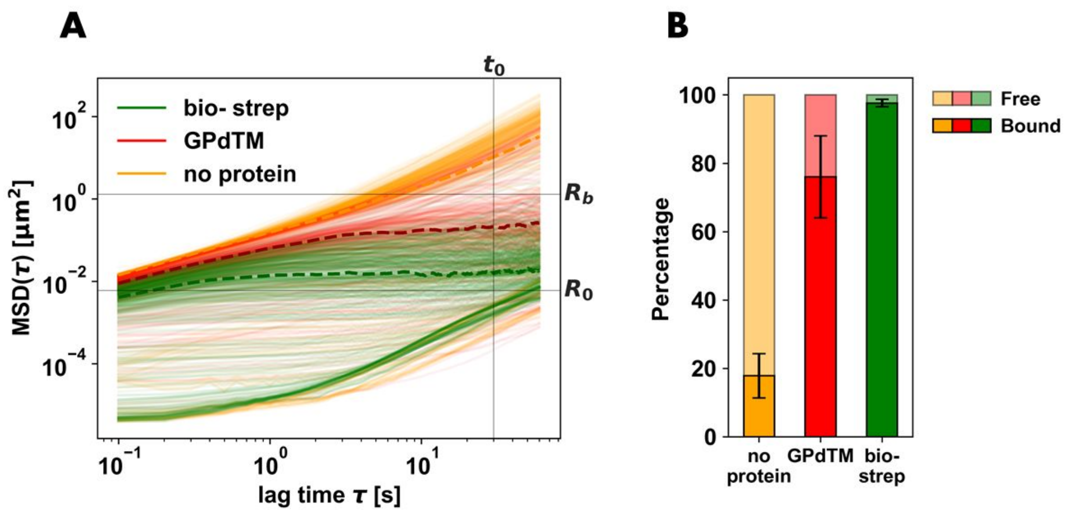

Figure S3: MSD and binding fractions for control experiments

A. A time average MSD plot of individual particles in three experimental conditions: In the absence of GPdTM (yellow), in the presence of GPdTM (red), and in the absence of GPdTM on a membrane incorporating biotinylated lipids (green). Dashed lines represent the MSD of the median particle (determined by its MSD at t_0): In absence of GPdTM $MSD(t_0) \pm CI\ 95\% = 10.4 \pm 3.2\ \mu m^2$; In presence of GPdTM, $0.2 \pm 0.06\ \mu m^2$; Biotinylated lipids $0.02 \pm 0.003\ \mu m^2$. The non-linear increase in MSDs of free and stuck particles is a result of the slow drift affecting the liquid and the membrane, respectively. **B.** The calculated percentage of bound and free particles for each of the corresponding conditions. Error bars represent ± SEM. In absence of GPdTM 17.9 ± 6.5 %; In presence of GPdTM, 76.0 ± 12.0 %; Biotinylated lipids: 97.6 ± 1.1 %. The statistical significance for the GPdTM and biotinylated lipids samples, determined by the nonparametric Mann-Whitney test, was $p = 0.0080$ and $p = 0.0001$, respectively.

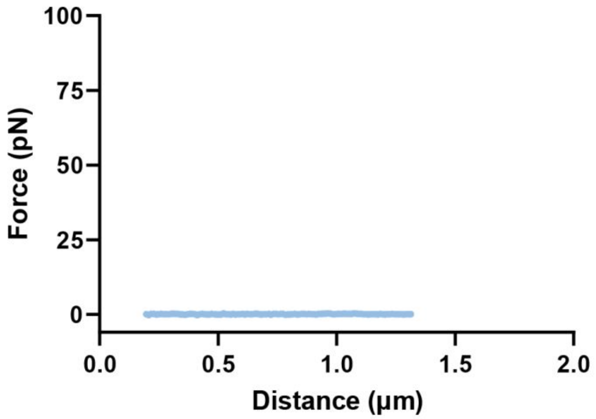

Figure S4: Single-molecule experiment measurement

A representative plot of an approximation and separation cycle with no interaction between the two microspheres.

Table S1: Single particle tracking results for all conditions presented in Figure 2**.**

The top part corresponds to Figure 2A, and the bottom part to Figure 2B. Columns represent the calculated mean fraction of bound particles, its SEM, the number of measurements used in the calculation, and the average number of particles in each measurement.

Condition	Fraction of Bound Particles (%)	SEM	n	Mean # particles
no GPdTM	29.61	±2.56	6	65
pH 5.2, Ca	89.12	±0.83	6	67
pH 5.2, EDTA	87.12	±2.71	6	72
pH 6.3, Ca	37.56	±5.94	4	69
pH 6.3, EDTA	36.04	±2.22	4	67
pH 7.5, Ca	27.06	±5.65	6	72
pH 7.5, EDTA	22.81	±1.92	6	66

Condition	Fraction of Bound Particles (%)	SEM	n	Mean # particles
GPdTM – sNPC1	54.04	±4.88	3	77
GPdTM + sNPC1	59.96	±5.22	3	60
